# Relapsed boyhood tibia polymicrobial osteomyelitis linked to dermatophytosis: a case report

**DOI:** 10.1186/s12893-022-01600-4

**Published:** 2022-05-04

**Authors:** Ping Kong, Youliang Ren, Jin Yang, Wei Fu, Ziming Liu, Zhengdao Li, Wenbin He, Yunying Wang, Zhonghui Zheng, Muliang Ding, Edward M. Schwarz, Zhongliang Deng, Chao Xie

**Affiliations:** 1 Department of Orthopaedics, Affiliated Hospital of Zunyi Medical University, Zunyi, 563000 China; 2 Joint Orthopaedic Research Center of Zunyi Medical University and University of Rochester Medical Center, Affiliated Hospital of Zunyi Medical University, Zunyi, 563000 China; 3 Department of Orthopaedics, Second Affiliated Hospital of Chongqing Medical University, Chongqing, 400065 China; 4 Department of Orthopaedics, Guizhou Provincial People’s Hospital, Guiyang, 550000 China; 5 Institute of Sports Medicine Beijing Key Laboratory of Sports Injuries Peking University Third Hospital, Beijing, 100191 China; 6 Department of Orthopaedics, First People’s Hospital of Xuzhou, Affiliated Hospital of China University of Mining and Technology, Xuzhou, 221005 China; 7 Department of Trauma, Shanghai East Hospital, Tongji University School of Medicine, Shanghai, 200065 China; 8 Department of Laboratory Medicine, Second Affiliated Hospital of Chongqing Medical University, Chongqing, 400065 China; 9 Department of Orthopaedics, Second Affiliated Hospital of Central South University, Changsha, 410008 China; 10 Department of Orthopaedics, Center for Musculoskeletal Research, University of Rochester Medical Center, Rochester, NY 14642 USA

**Keywords:** Polymicrobial osteomyelitis, *Staphylococcus aureus*, *Corynebacterium*, Dermatophytosis, Relapse

## Abstract

**Background:**

Relapsed childhood polymicrobial osteomyelitis associated with dermatophytosis has not been reported in the literature.

**Case presentation:**

Here we report on a case of a 45-year-old man who had left tibial osteomyelitis for 29 years, accompanied by skin fungal infection of the ipsilateral heel for 20 years, and underwent a second operation due to recurrence of polymicrobial infection 6 years ago. The patient had a history of injury from a rusty object, which penetrated the anterior skin of the left tibia middle segment causing subsequent bone infection, but was asymptomatic after receiving treatments in 1983. The patient was physically normal until dermatophytosis occurred on the ipsilateral heel skin in 1998. The patient complained that the dermatophytosis was gradually getting worse, and the tibial wound site became itchy, red, and swollen. The left tibial infection resurged in May 2012, leading to the patient receiving debridement and antibiotic treatment. H&E and Gram-stained histology was performed on biopsy specimens of sequestrum and surrounding inflammatory tissue. Tissue culture and microbiology examination confirmed polymicrobial infection with *Staphylococcus aureus* (*S. aureus*) and *Corynebacterium* and a fungus. Additionally, the patient also received potassium permanganate for dermatophytosis when he was admitted into the hospital.

**Conclusions:**

Together with longitudinal follow-up of medical history, surgical findings, histopathological and microbiology culture evidence, we conclude that boyhood tibia polymicrobial osteomyelitis with *S. aureus* and *Corynebacterium* occurred in this patient, and the fungal activation of dermatophytosis may have led to osteomyelitis relapse.

## Background

Osteomyelitis is a devastating disease caused by microbial infection of the bone, with recurring and persistent infections occurring in approximately 40% of patients [1, 2]. The recurrence of osteomyelitis may be related to many factors such as biofilm formation, intracellular infection and bacteria colonizing inside of osteocytic-canalicular network of live cortical bone. Since the most rigorous prophylactic and surgical procedures cannot reduce infection rates, it has become clear that the host’s state of immunity plays an essential role in orthopaedic infections [3, 4]. Thus, the 2018 international consensus meeting on musculoskeletal infection concluded that development of effective immunotherapy against osteomyelitis is among the highest priorities in orthopaedics [3, 5].

Dermatophytosis is a superficial fungal infection mostly restricted to keratinized tissues, which stimulates cell-mediated immune responses in the acute phase. However, these immune responses appear to be transient, as they are not present in patients who suffer from chronic or recurrent infections [6, 7]. Osteomyelitis is rarely caused by fungal infection [8, 9], and there are no documented cases of *Staphylococcus aureus (S. aureus) and corynebacterium* osteomyelitis whose recurrence was accompanied by dermatophytosis. Here we report such a case, and pose the possibility that immunomodulation from dermatophytosis has the potential to reactivate dormant *S. aureus* osteomyelitis to cause clinical relapse.

## Case presentation

A 45-year-old man had an injury caused by a rusty object leading to the infection of osteomyelitis of the left tibia at 10-year-old in 1983. He received therapy in June 1983 and recovered fully. Prior to 1998, there were no symptoms, but the patient complained that dermatophytosis on the left foot heel had occurred around 1998. Interestingly, since then, when the symptom of dermatophytosis worsened, an adjacent skin wound of the left tibia had appeared. The wound was painful, red, swollen and had pruritus. Prior to 2012, the patient only received irregular treatment at the community clinics and had not been suffering from localized pain, swelling and heat on the surgical sites for nearly 3 years. In May 2012, the left heel dermatophytosis worsened dramatically, as the surgical site of the left tibia presented with marked pain, swelling and heating for about 10 days, which prompted his hospital admission on May 17, 2012.

**Fig. 1:**
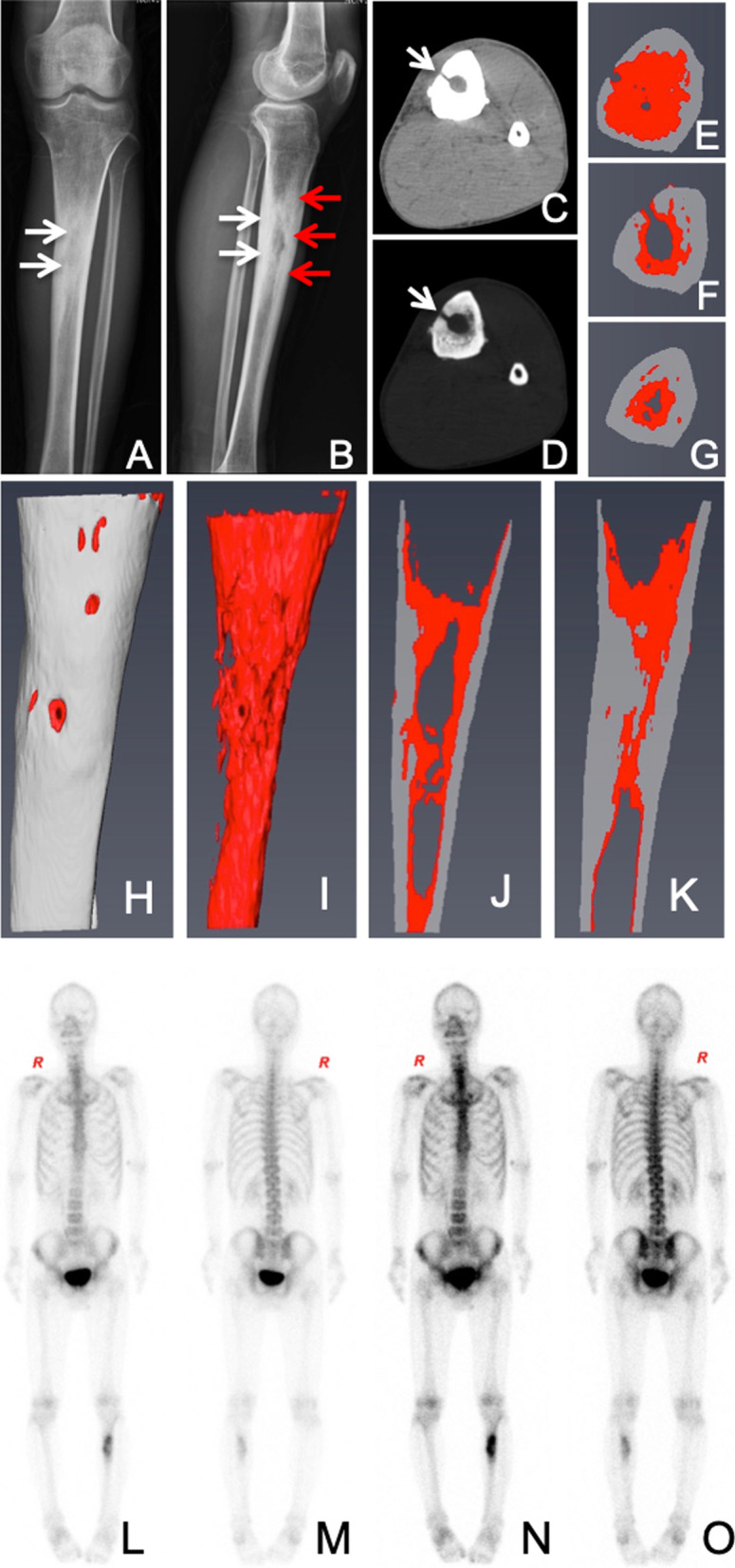
Preoperative imaging evaluation determined osteomyelitis lesion range and severity in 2012. The plain radiography of left tibial approximal diaphysis showed a central area of radiolucency with a surrounding thick rim of reactive bone sclerosis (**A**, **B** white arrows). CT scan found a cloaca (**C**, **D** white arrows) and adjacent reactive bone (**E**–**G**, red areas) from lesion area of Panel B (red arrows). Then we performed 3D reconstruction analysis by using a software Amria to determine the reactive bone within the intramedullary (**H** tibial; **I** intramedullary reactive bone; **J**, **K** sagittal section of tibia). The infection was confirmed by the emission computed tomography (ECT) (**L**–**O**)

After admission, there were no other complications or comorbidities. The body mass index (BMI) was 18.0, the vital signs were T 36.8 °C, P 80/min, R 20/min, and BP 95/62 mmHg (12.67/8.27 kPa). The initial physical examination indicated that there was a 3.0 cm × 3.0 cm subcutaneous fluctuation in the anteromedial diaphysis of the left tibia, and the local skin temperature was high. A peripheral blood test showed an erythrocyte sedimentation rate (ESR) of 42 mm/h, C-reactive protein (CRP) 16 mg/L, and WBC 5.79/mm^3^ with 64.5% granulocytes. Plain radiography scans, computed tomography scans (CT) and emission computed tomography scans (ECT) were all performed (Fig. 1). All three scans revealed active bone infection and reactive ectopic bone formation. A 3D visualization and analysis software Amira (Thermo-Fisher Scientific, Waltham, MA, USA) was employed (Fig. 1C–K), which showed there was a sequestrum in the medullary cavity and adjacent reactive bone formation. Further ECT scans indicated that the localized area of uptake had increased (Fig. 1L–O).

**Fig. 2:**
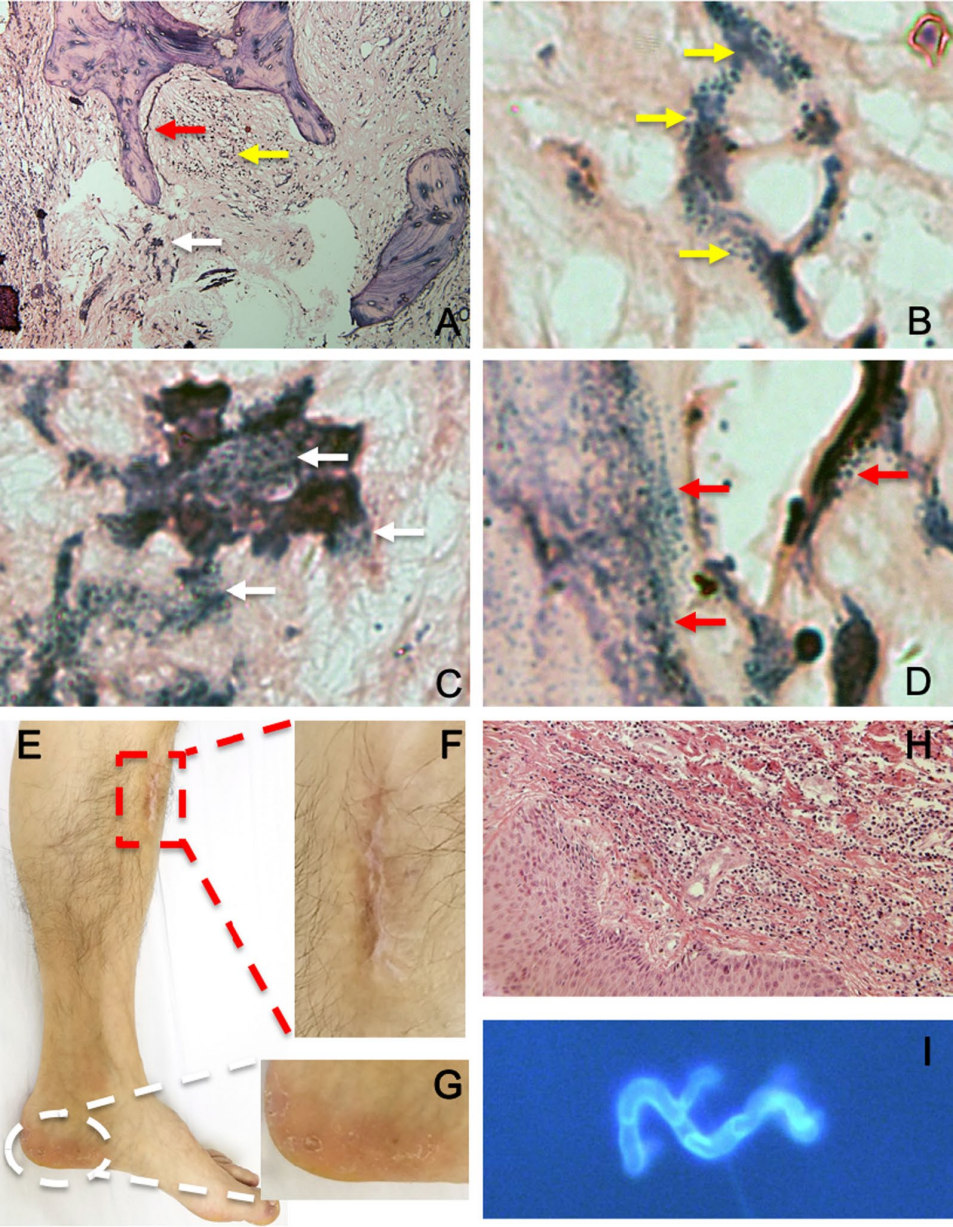
Polymicrobial tibia infection confirmed by histopathological debrided sequestrum, and dermatophytosis confirmed by biopsy. The sequestrum (**A**–**D**) and surrounding inflammatory tissue (**H**) were collected during operation debridement, and Gram staining was performed (**A**) to show Gram-Positive staining in soft tissue (Yellow arrowheads) (**A**, **B**), and sequestrum bone (white and red arrowheads) (**A**, **C**, **D**), but adjacent skin shown chronic inflammatory cells and neutrophil infiltration (**H**) in 2012. The fellow-up (**E**–**G**) fungal culture-positive (**I**) from left heel skin biopsy in 2018

**Fig. 3:**
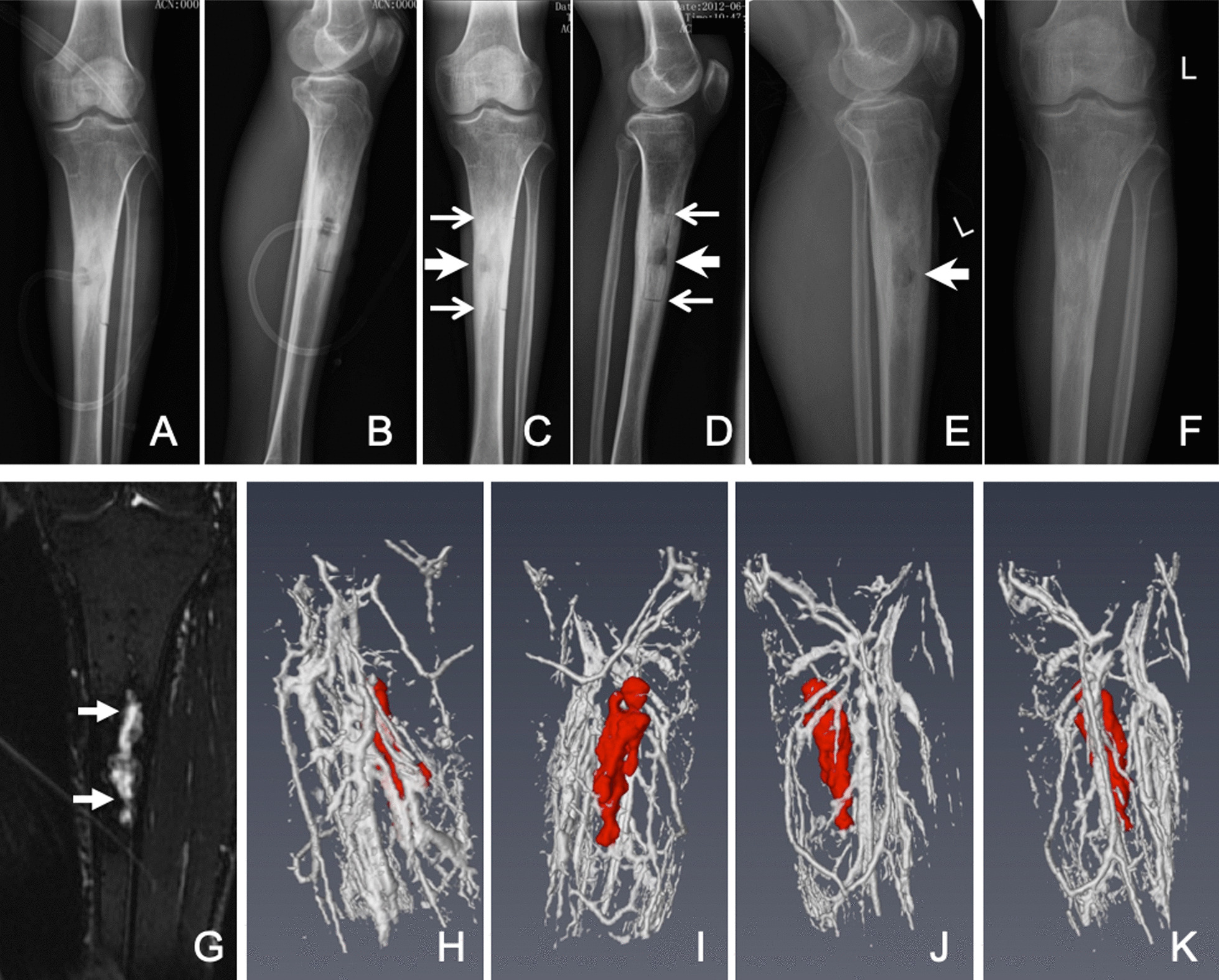
Long-term follow-up shown limited tibial fistulous tract still remaining. The post operation follow-up were performed by plain radiography on May 25th (**A**, **B**) and June 18th, 2012 (**C**, **D**); and April 16th, 2018 (**E**, **F**), and MRI on April 24th, 2018 (**G**–**K**). There is an open window for decompression drainage on the left tibial anteromedial side (**A**–**D**) which gradually healed and recanalization of the medullary cavity (**E**, **F**). Although the bone infection lesions were not able to completely removed (**G**: white arrows, red segmentations in **H**–**K**), the abundant blood supply around the lesions limited the spread of the infection lesions to the surrounding shown in MRI 3D reconstruction (**H**–**K**)

During admission, the patient received surgical debridement and vacuum sealing drainage (VSD) three times (May 21st, 28th and Jun 4th, 2012). For the first operation, the histopathological specimen of infected bone and soft tissues were collected from the wound of upper anteromedial diaphysis for Gram staining (Fig. 2A–D) and H&E staining (Fig. 2H). The bacterial cultures were positive for *Corynebacterium* and a methicillin-susceptible strain if *S. aureus* resistant to penicillin, erythrocin, amoxicillin, clarithromycin, tetracycline, and roxithromycin. Prior to these results of the antibiotic susceptibility test by the medical lab, the patient received empirical combinations of antimicrobial therapy sequentially with ceftizoxime (30 days) and piperacillin zobartan (35 days), which was the right combinations that were confirmed by the bacterial culture and antibiotic susceptibility test. Sealing of the drainage at different times occurred over a period of 12 days. Additionally, on 13th day of the admission, he received potassium permanganate tablets for dermatophytosis that lasted 3 days. Postoperative re-examination X-rays were performed on May 21, 2012 (Fig. 3A, B) and Jun 18, 2012 (Fig. 3C, D) respectively.

After discharge on Jun 29, 2012, the surgical sites of the left low limb recovered well. Besides his normal activities, the patient could play badminton nearly five times a week during the following 3 years. However, the patient complained of recurrent fungal infection of the left heel in 2015, with associated pathology in the left anteromedial tibia that presented as itching, redness, swelling, heat and pain. The patient noticed there was a positive correlation between the two sceneries, which was that when the foot fungal infection worsened, the osteomyelitis lesion became aggravated as indicated above. These symptoms recurred intermittently lasting about 3 more years.

The patient came for a follow-up in April 2018. Other than the two surgical scars, the appearance of the left low limb was not observed to be significantly abnormal (Fig. 2E, F). Radiological examinations (Fig. 3E, F) and magnetic resonance angiography (MRA) (Fig. 3G–K) of the left low limb were also performed. The MRA 3-D reconstruction showed that abundant vasculature formed around the lesions and debridement site, and suggested the blood supply was well established (Fig. 3H–K). Additionally, the skin lesions on the left heel are visible, and a smear was sampled for fungal culture (Fig. 2I). The immune globulins of peripheral blood were examined for IgG (13.63 g/L), IgA (1.45 g/L), and IgM (0.83 g/L), which were in their normal ranges, respectively.

## Discussion and conclusions

*S aureus* is responsible for the majority of osteomyelitis cases [2, 10]. It has multiple microbial surface components recognizing adhesive matrix molecules and cell wall-anchored proteins important for the pathogenicity of infection [1]. Notably, collagen adhesion protein (Cna) and bone sialoprotein binding protein (Bbp) favor fibronectin-binding proteins (FnBP) internalize into nonprofessional phagocytic cells [11]. This internalization of persisting intracellularly as a small-colony variant (SCVs), along with glycocalyx formation on implant hardware and necrotic tissue, and colonization of the osteocyte-lacuno canalicular network (OLCN) of cortical bone are the three distinct reservoirs of bacterial biofilm, which are the dominant cause of chronic osteomyelitis recurrence [1, 10, 12]. Of note, *S. aureus* in a biofilm can display variable growth rates, altered oxygen, and nutrient dependence and acquired virulence mechanisms via horizontal gene transfer [13]. Knowing that a single clonal strain of *S. aureus* can remain quiescent for decades within the patient without clinical symptoms has been well-established [14, 15].

Naturally, *S. aureus* competitively interacts with other bacterial species. *S. aureus* and *Corynebacterium* are the two most important species infecting the skin and nasopharynx. Both species utilize similar competitive strategies for the same adhesion site with host epithelial cells, and *S. aureus* secretes bacteriocins actively against *Corynebacterium* [16, 17]. However, polymicrobial bone infection of *S. aureus* with *Corynebacterium* has yet to be documented. *Corynebacterium* species are gram-positive, catalase-positive, aerobic or facultatively anaerobic microorganisms of the human skin and mucous membranes that were originally thought to be contaminants, but are now recognized as pathogenic species [18]. A few cases were reported as *Corynebacterium* relapses of prosthetic joint infections (PJIs), which were mostly associated with non-optimal surgical management, and only one case of *Corynebacterium* osteomyelitis in an immunocompromised child was reported [19, 20]. Unfortunately we did not find the patient’s original medical records of 1983, and were unsucceful performing DNA extraction from paraffin tissues to identify the bacterial whole genome sequencing of 2012. Thus, there is no direct laboratory evidence to prove that the pathogenic bacterium in 2012 is the same as the one in 1983. However, based on the specimen for the pathological exam collected from surgical debridement bone tissue directly, we speculate that the pathogens that caused the polymicrobial osteomyelitis are the same ones.

Dermatophytosis is a superficial fungal infection mostly restricted to keratinized tissues. Interaction between the host immune system and the fungal cell initiates with the innate immunity as keratinocytes directly participate in defense against the pathogen. This involves pattern-recognition receptors (PRR) and C-type lectin receptors (CLR) with Toll-like receptors (TLR). The PRRs are expressed on host macrophages, dendritic cells, keratinocytes and neutrophils, which release proinflammatory cytokines interleukin (IL) (IL-1β, IL-6 and IL-17), chemokines (IL-8, MCP-1, eotaxin and eotax- in-2) and immunomodulatory lymphokines (IL-4 and IL-13) [6, 21]. These factors further stimulate T- and B-lymphocyte proliferation to produce immunoglobulins (Ig). Thus, the status of host immune function is indicated by the level of antigen specific immunoglobins such as IgG, IgA, IgM. Although osteomyelitis is rarely caused by the fungus, Menon et al. reported two cases of ribs infection, and Sidhu et al. published 22 cases of co-infective bacterial and fungal in prosthetic joint infections, and the most of these individuals were under immunocompromised condition [8, 9]. Additionally, Conway and collages found that patients with orthopedic infection using serologic markers to quantify the competence of their immune system while actively infected, the normal IgG levels were considered abnormal when infection was present [4]. While we have no direct evidence to prove that the heel dermatophytosis in this patient reactivated the polymicrobial osteomyelitis of *S. aureus* and *Corynebacterium*, this is suggested by the timing of the fungal infection on left heel in 2008 and recurrent in 2015 was followed by lesions of the left anteromedial tibia infection symptom. This suggests that the recovery or recurrence of heel fungal infection could be regarded as a monitor of the patient’s immune function level, which can indirectly indicate the early warning signal of whether there is a risk of recurrence of tibial osteomyelitis.

In summary, this case shows that polymicrobial osteomyelitis of *S. aureus* with *Corynebacterium* can reoccur after remaining dormant or quiescent for many years from childhood to adult life, and that fungal dermatophytosis of the heel may induce immune modulation that results in relapse of osteomyelitis.

## Data Availability

The dataset supporting the conclusions of this article is included within the article.

## Abbreviations

BMI: Body mass index
T: Temperature
P: Pulse
R: Respiration
BP: Blood pressure
ESR: Erythrocyte sedimentation rate
CRP: C-reactive protein
WBC: White blood cells
CT: Computed tomography
ECT: Emission computed tomography
VSD: Vacuum sealing drainage
Cna: Collagen adhesion protein
Bbp: Bone sialoprotein binding protein
FnBP: Favor fibronectin binding proteins
SCVs: Small-colony variants
